# Privacy-Preserving Identity-Based Undeniable Signatures from Lattices with Controlled Verifiability for IoT Networks

**DOI:** 10.3390/s26196205

**Published:** 2026-09-30

**Authors:** Jing Cheng, Rui Lu, Chaoyang Li, Haseeb Ahmad, Shujun Liang

**Affiliations:** 1 Engineering Training Center, Zhengzhou University of Light Industry, Zhengzhou 450001, China; chengjing@zzuli.edu.cn; 2 College of Software Engineering, Zhengzhou University of Light Industry, Zhengzhou 450001, Chinalichaoyang@zzuli.edu.cn (C.L.); 3 Department of Computer Science, National Textile University, Faisalabad 37610, Pakistan; 4 Research Institute of Industrial Technology, Zhengzhou University of Light Industry, Zhengzhou 450001, China

**Keywords:** IoT network, identity, undeniable signature, lattice cryptography

## Abstract

The Internet of Things (IoT) network plays an important role in production and people’s daily life. IoT networks generally face security problems of privacy leakage and message denial. To protect the privacy of IoT data and users, this paper first introduces a controlled verification model for IoT networks and then proposes an identity-based undeniable signature (ID-US) scheme. This controlled verification model establishes a secure IoT data-sharing channel with blockchain technology, which reduces data tampering and inconsistency. This ID-US scheme is constructed with lattice cryptography, which can provide strong resistance to quantum attacks. Meanwhile, the undeniable and identity mechanisms ensure that the signed message cannot be denied. Next, the security proof in the random oracle model shows that this ID-US can achieve soundness and unforgeability. The Scyther test results show that there are no attacks by verifying 11 points of attacks from the perspectives of the KGC, signer, and verifier. Moreover, the efficiency analysis and performance analysis of the key size, time consumption, energy consumption, and transaction latency have been performed, and the results show that the ID-US scheme is more efficient than similar schemes in the recently published literature, and the controlled verification model can protect IoT data privacy and support secure IoT data sharing with the undeniable property.

## 1. Introduction

The rapid proliferation of the IoT network has reshaped the way physical devices, sensing systems, and back-end cloud services interact, giving rise to large-scale, heterogeneous networks that span smart homes, industrial control systems, vehicular ad hoc networks, healthcare monitoring, and smart-city infrastructures [1]. Recent industry estimates project the number of deployed IoT endpoints to exceed 29 billion by 2030, and the data generated by these devices is increasingly mission-critical: it drives automated decision-making, machine-learning analytics, and even safety-relevant control loops [2]. Consequently, ensuring the confidentiality, integrity, authenticity, and non-repudiation of IoT data has become a first-order concern for both academia and industry.

At the same time, the open and pervasive nature of IoT deployments creates an unprecedented attack surface. Adversaries may eavesdrop on wireless channels, impersonate legitimate nodes, inject forged sensing data, or repudiate previously issued commands. Traditional security mechanisms, in particular public-key digital signatures, are therefore widely recognized as a fundamental building block of IoT security [3]. A valid signature attached to a message can be checked by any verifier and serves as cryptographic evidence that the message originated from the claimed sender and has not been altered in transit, providing the integrity and non-repudiation properties required for many IoT applications [4], including firmware updates, over-the-air software provisioning, and cross-domain data exchange. However, classical signature schemes do not, by themselves, guarantee privacy. In conventional public-key infrastructures (PKI), a signature is a publicly verifiable object: anyone holding the signer’s public key can verify it at any time, without the signer’s consent. While this is desirable in open systems, it has serious consequences in privacy-sensitive IoT scenarios. Consider a wearable health monitor that periodically signs a patient’s heart rate and transmits the signed report to a hospital cloud. Under a standard signature scheme, any party that intercepts the signed report can verify its origin, link all reports produced by the same device, and thereby profile the patient’s daily activities, location patterns, and medical conditions. Similarly, in smart metering, vehicular networks, and location-based services, signed messages can be exploited as user identifiers or pseudonyms, eroding the very anonymity the IoT system is meant to preserve [5]. This tension between public verifiability and user privacy is one of the central challenges in designing signature schemes for modern IoT environments.

To reconcile these two requirements, undeniable signature schemes can offer an elegant primitive [6]. In an undeniable signature scheme, a signature is not publicly verifiable; instead, verification is carried out through an interactive confirm/deny protocol between the signer and the verifier, so that the validity of a signature can only be established with the cooperation of the signer. This selective verifiability provides a natural mechanism for content protection and signer privacy: signatures cannot be duplicated, widely disseminated, or used to track users without the signer’s consent. As a result, undeniable signatures have attracted considerable attention in privacy-preserving applications such as electronic voting, licensed software distribution, and secure electronic health record sharing [7,8,9]. A parallel line of research seeks to simplify key management in large-scale IoT deployments. Classical PKI requires every user to obtain a certificate binding their identity to a public key, which is cumbersome for billions of resource-constrained devices [10]. Identity-based cryptography (IBC) eliminates the need for certificates by deriving a user’s public key directly from a publicly known identifier [11]. A trusted Key Generation Center (KGC) issues the corresponding private key. The pairing-based construction, in particular, has inspired a rich body of identity-based signature (IBS) schemes [4,12,13], and a number of identity-based undeniable signature (ID-US) schemes [14,15], which combine the certificate-free convenience of IBC with the selective verifiability of undeniable signatures and are particularly well suited to IoT.

Despite their appealing functionality, nearly all existing ID-US schemes are built on the hardness of number-theoretic problems such as the discrete logarithm problem (DLP), the integer factorization problem (IFP), or the computational/decisional Diffie–Hellman problem (CDH/DDH) in finite groups or pairing-friendly elliptic curves [16]. The advent of large-scale quantum computers, however, fundamentally threatens this foundation: Shor’s algorithm solves DLP, IFP, and CDH in polynomial time [17], rendering all pairing-based and RSA-type schemes insecure. With the National Institute of Standards and Technology (NIST) actively standardizing post-quantum primitives and the “harvest-now, decrypt-later” threat already materializing in IoT deployments with long secrecy lifetimes, it is essential that the cryptographic core of IoT security be quantum-resistant.

Lattice-based cryptography has emerged as one of the most promising post-quantum paradigms, offering several compelling advantages for IoT [18]: (i) worst-case to average-case hardness reductions under standard lattice assumptions such as the Short Integer Solution (SIS) and Learning With Errors (LWE) problems; (ii) resistance to known quantum attacks; (iii) relatively efficient arithmetic over small integers, which is attractive for resource-constrained sensors and RFID-class devices; and (iv) a rich algebraic structure that supports advanced functionalities, including identity-based encryption, attribute-based encryption, and digital signatures. The NIST Post-Quantum Cryptography Standardization Process has further validated the maturity of lattice constructions, with CRYSTALS-Dilithium [19] and Falcon [20] selected as primary digital signature standards. Nevertheless, despite these advances, the literature on lattice-based identity-based undeniable signatures remains surprisingly sparse. The few existing works either rely on the pre-image sampleable trapdoor paradigm without addressing confirm/deny protocols, or build on weaker non-standard assumptions, leaving a significant gap in provably secure, post-quantum, privacy-preserving IoT authentication.

Motivated by these observations, a lattice-based identity-based undeniable signature (ID-US) scheme is specifically tailored to the privacy, efficiency, and security requirements of IoT networks. The main contributions are summarized as follows:

- A controlled verification model is introduced based on blockchain for IoT networks. IoT data is collected by the edge IoT devices, and then the data-sharing processes are protected by an ID-US scheme and transformed through a distributed blockchain network. This mechanism solves the privacy leakage and IoT data loss problems.
- An ID-US scheme is proposed to support the controlled verification model. This ID-US integrates identity and undeniable mechanisms to achieve user anonymity, data anonymity, and signature non-repudiation. Meanwhile, this ID-US scheme is constructed with the lattice assumption, which provides anti-quantum-attack security for IoT networks.
- The security proof of the ID-US scheme in the random oracle model is presented. Some related security analyses are presented, and the security analysis results show that this ID-US scheme is correct and secure.
- The comparison and performance have been executed. The security test with Scyther has been executed, and the results show that there are no attack weak points. The efficiency comparison of key size and time consumption is presented. The performance evaluations are also simulated. The results show that this ID-US scheme is efficient. Meanwhile, the transaction latency has been compared. The comparison results show that this controlled verification model provides enhanced security while ensuring low latency.

In the following, Section 2 reviews IoT network privacy protection and post-quantum privacy protection methods. Section 3 presents the lattice theory. Section 4 introduces the controlled verification model and ID-US scheme. Section 5 gives the security analysis of the ID-US scheme and tests it with Scyther. Section 6 evaluates its efficiency and performance. Finally, Section 7 shows the conclusion.

## 2. Related Work

This section reviews prior work along two complementary axes. Section 2.1 surveys network-level privacy protection mechanisms that have been proposed for IoT, and Section 2.2 then narrows the focus to post-quantum privacy-preserving techniques for IoT.

### 2.1. IoT Network Privacy Protection

Pseudonym-based schemes replace long-lived identifiers with short-lived pseudonyms that are rotated periodically to break linkability across sessions. Such schemes have been widely studied in VANETs, smart metering, and wearable health monitoring [21,22]. Pseudonym schemes are simple and compatible with existing PKI or IBS infrastructure. However, they offer only weak privacy, because they rely on frequent synchronous rotation and remain vulnerable to side-channel deanonymization through timing and RF fingerprinting.

Group and ring signatures allow a member of an authorized group to produce a signature that is verifiable as originating from some member of the group while concealing the actual signer. They have been applied to smart-grid billing, anonymous sensor-data credentialing, and vehicular announcements [23,24]. Attribute-based signatures (ABS) and attribute-based encryption (ABE) bind keys and signatures to descriptive attributes instead of explicit identities. They support fine-grained, policy-driven access control in healthcare and industrial IoT [25,26]. Group and ring signatures provide strong in-group anonymity and support dynamic revocation, yet they depend on a trusted group manager who can deanonymize any signature, and ring signatures incur sizes that grow linearly with the ring size, which is costly on bandwidth-limited IoT links. Attribute-based schemes enable fine-grained access control and hide identities behind attribute sets, but most efficient constructions rely on pairings. Such schemes are expensive on IoT-class hardware without dedicated pairing accelerators and are vulnerable to quantum attacks.

Complementary to the above, homomorphic encryption enables cloud aggregators to compute over encrypted IoT readings [27,28]. Differential privacy adds calibrated noise to aggregate outputs in order to bound the influence of any single record [29]. Blockchain-based trust management removes single trusted authorities by anchoring identities and signatures on a tamper-evident ledger, often combined with ring signatures or zero-knowledge proofs [30]. Homomorphic encryption and differential privacy offer strong cryptographic or information-theoretic guarantees for aggregated IoT data, but neither of them provides per-message authentication or non-repudiation. Such guarantees are essential for safety-critical IoT commands and signed alerts. Fully homomorphic encryption is still far too expensive for typical IoT workloads. Blockchain-based architectures eliminate single points of failure, yet on-chain transactions incur significant latency, throughput, and energy overheads that conflict with IoT bandwidth and battery constraints. Meanwhile, public ledgers can themselves become a privacy liability when off-chain identities are linked to on-chain addresses. In addition, quantum methods, such as quantum key distribution and quantum Internet, have been introduced to enhance network security for IoT applications [31,32].

In summary, no single family from the IoT privacy literature delivers signer anonymity, signature non-repudiation, and quantum resistance at the same time. The primitive that comes closest in functionality, namely undeniable signatures, has not yet been built on a post-quantum foundation.

### 2.2. Post-Quantum Privacy Protection Methods

The NIST post-quantum standardization process has stimulated a rich body of work on quantum-resistant IoT security. Lattices are currently regarded as the most mature family for signatures [18]. They offer multiple standardized primitives, well-studied implementations, and a rich toolkit of advanced functionalities including IBE, ABE, ABS, and group signatures.

Modern lattice cryptography rests on LWE and SIS. Both admit worst-case to average-case reductions from standard lattice problems. Their algebraic variants, Ring-LWE and Module-LWE, enable compact representations and small keys and signatures, while preserving the worst-case guarantees. Building on these tools, a series of provably secure lattice signature schemes has been proposed. Combining identity-based cryptography with lattice foundations yields lattice-based identity-based signatures (L-IBS) [33,34,35]. Such schemes remove certificates while remaining quantum-resistant. A small but growing line of work has attempted to construct lattice-based undeniable signatures [8,9,14,15,36,37]. These constructions combine selective verifiability with post-quantum security and represent the most direct precursors of our scheme. Lattice foundations are attractive because they offer worst-case to average-case hardness, resistance to known quantum attacks, and arithmetic over small integers suitable for resource-constrained sensors and RFID-class devices. Their main drawbacks are larger signature and key sizes compared to RSA/ECDSA at the same classical security level and the limited number of side-channel-hardened implementations. In summary, no prior scheme simultaneously achieves post-quantum security under standard lattice assumptions, identity-based key generation, full confirm/deny selective verifiability, and strong anonymity with a rigorous invisibility proof.

The former two subsections together reveal a common gap in the literature. However, none of these families simultaneously guarantees signer anonymity, signature non-repudiation, and quantum resistance. Meanwhile, the post-quantum privacy literature lacks a construction that integrates undeniable selective verifiability with identity-based key generation and rigorous invisibility proofs. In this work, a lattice-based ID-US scheme is proposed to cover these challenges.

## 3. Preliminaries

Some definitions of lattice theory are presented.

**Definition** **1.** ***Lattice [38]**: $v_{1},\cdots ,v_{n}\in R^{m}$ is a set of linearly independent vectors; the lattice $\Lambda_{L}$ is shown in Equation (1):*

(1) $$\Lambda_{L}=\left\{a_{1}v_{1}+a_{2}v_{2}+\cdots +a_{n}v_{n}:a_{1},a_{2},\cdots ,a_{n}\in Z\right\}$$

*where $A=\left(a_{1},\cdots ,a_{m}\right)\subset R^{n\times m}$ is a basis of lattice* Λ*. Here, n is the dimension and m is the rank, which satisfy m=O(nlogq).*

**Definition** **2.** ***q-ary Lattice [38]*** *: The “q-ary” lattice is shown in Equation (2), where q is a prime number and $A\in Z_{q}^{n\times m}$ is a matrix.*

(2) $$\begin{array}{cc} \; & L^{⊥}\left(A\right)=\left\{x\in Z^{m}|Ax=0\;modq\right\}\\ \; & L_{y}^{⊥}\left(A\right)=\left\{x\in Z^{m}|A^{T}y=x\;mod\;q\;for\;y\in Z^{n}\right\} \end{array}$$

**Definition** **3.** ***Gaussian distribution [39]*** *: With standard deviation σ∈R and center c∈R evaluated at x∈R, the Gaussian distribution is defined by $\rho_{c,\sigma }\left(x\right)=exp\left(\frac{-{\left(x-c\right)}^{2}}{2\sigma^{2}}\right)$, and more generally by $\rho_{c,\sigma }\left(x\right)=exp\left(\frac{-||x-{c||}^{2}}{2\sigma^{2}}\right)$ for $x,c\in R^{n}$. Here, $\rho_{\sigma }\left(x\right)$ represents the Gaussian distribution when the center c=0. $D_{\sigma }\left(x\right)=\rho_{\sigma }\left(x\right)/\rho_{\sigma }\left(Z\right)$ represents the discrete Gaussian distribution over Z with center c=0. $D_{\sigma }\left(x\right)=\rho_{\sigma }\left(x\right)/\rho_{\sigma }\left(Z^{m}\right)$ represents the more general situation over $Z^{m}$ with center c=0.*

**Definition** **4.** *$ℜ-{\mathrm{SIS}}_{q,n,m,\beta }^{\kappa }$* ***problem*** *[39]: Given a ring ℜ and a distribution κ over ${ℜ}_{q}^{n*m}$, $ℜ-SIS_{q,n,m,\beta }^{\kappa }$ is a problem to find non-zero $v\in {ℜ}_{q}^{m}$ to achieve the following Equation (3).*

(3) Av=0

*where $A\in {ℜ}_{q}^{n*m}$, and ${\left||v|\right|}_{2}\leq \beta$.*

**Definition** **5.** ***SampleMat algorithm [40]**: For the matrix $A\in Z_{q}^{n\times m}$ and the short basis $T\in Z_{q}^{m\times m}$ obtained by the* TrapGen(n,m,q) *algorithm, the secure Gaussian parameter $\sigma \geq \left(∥B∥\right)\cdot \omega \left(\sqrt{log_{2}n}\right)$ and the uniform matrix $U\in Z_{q}^{n\times k}$, this algorithm outputs a random matrix $S\in Z_{q}^{m\times k}$, where AS=0modq.*

## 4. Controlled Verification Model for IoT Network

A controlled verification model is presented first, and then an ID-US scheme based on the lattice assumption is proposed in this section.

### 4.1. Controlled Verification Model

**Figure 1:**
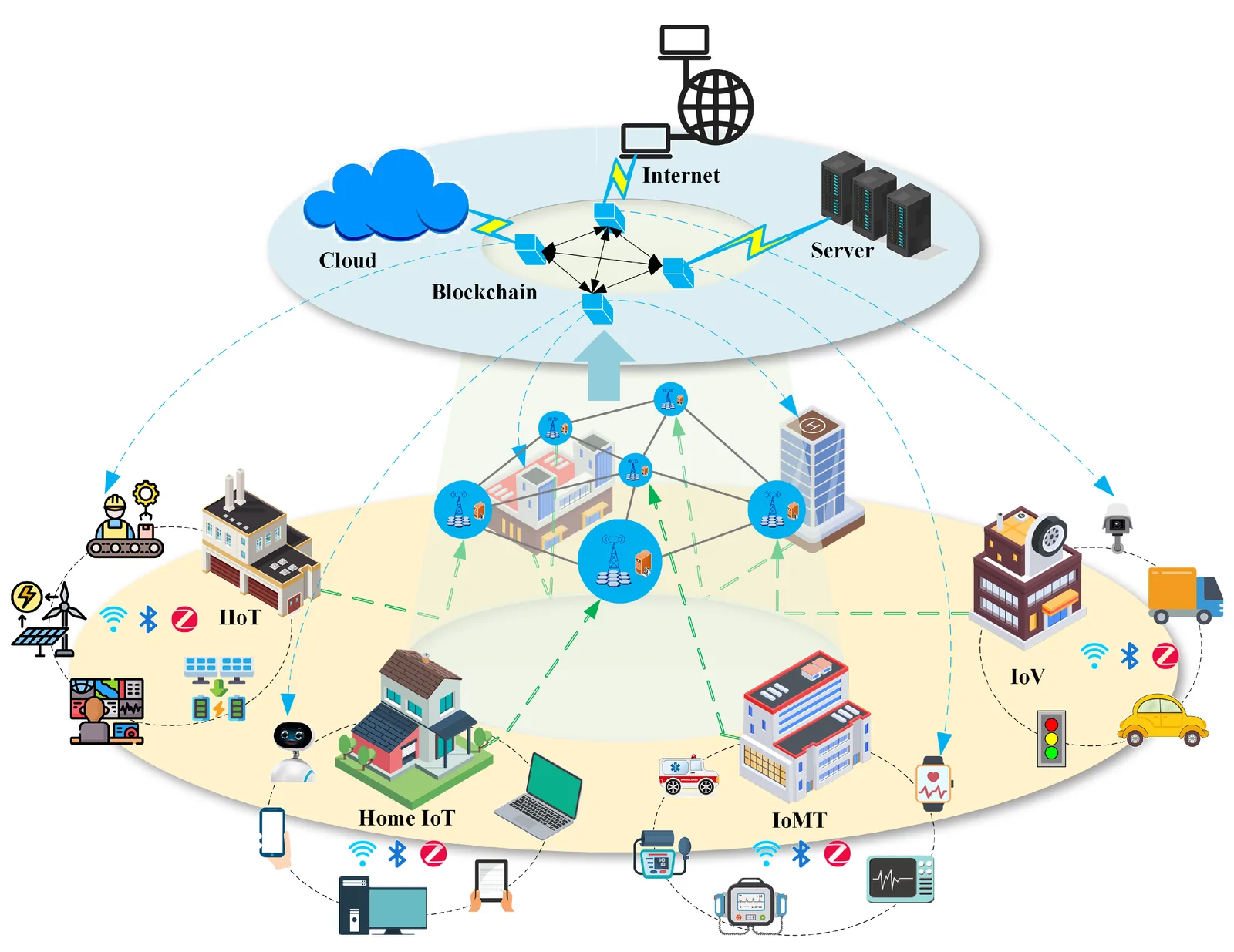
Controlled verification model.

Figure 1 illustrates a secure controlled verification model architecture for the multi-scenario Internet of Things. This model uses the ID-US scheme as its core. It seamlessly connects industrial, home, medical, and vehicular terminals to the cloud and blockchain. This architecture builds a complete closed loop across the data lifecycle. The loop covers data collection, transmission, storage, and utilization. The signature mechanism runs through the entire process. It ensures the authenticity, privacy, security, and non-repudiation of massive data throughout the lifecycle. This breaks traditional centralized trust bottlenecks. It creates a highly credible digital ecosystem.

- *Data Collection*: These scenarios include sensors and robots in industrial plants. They also include smart homes and personal terminals. Furthermore, they include wearable monitoring instruments for patients. Finally, they involve smart vehicles and traffic facilities on roads. Devices continuously generate massive amounts of environmental, production, physiological, and traffic data within this heterogeneous edge node network. The system allocates a unique identity-based key to every legal data collection terminal by introducing the ID-US scheme in this phase. Every device digitally signs data at the source during the collection process. This guarantees the native confidentiality of the data. It prevents unauthorized forged nodes from accessing the network and injecting false data. It fundamentally ensures clear, authentic, and unforgeable identity attribution for every bit of collected data. This lays a solid trust foundation for subsequent data flow.
- *Data Transmission*: This IoT network efficiently and stably relays data collected by edge terminals to the upper control center. The open wireless network plays an essential role in IoT data transmission. To prevent eavesdropping, interception, and man-in-the-middle tampering attacks, blockchain technology takes responsibility for transaction integrity, tamper-proof records, and network-wide consistency. Meanwhile, highly sensitive data is routed between base stations. This data includes medical signs, industrial commands, and vehicle collision warnings. The signature verification mechanism ensures the integrity of data packets in the channel. Any third party might attempt to tamper with or hijack data mid-transmission. This will cause signature verification to fail. IoT network nodes will immediately intercept and discard the altered data. This builds an indestructible data channel for applications highly dependent on real-time security.
- *Data Storage*: Data enters the storage stage after safely arriving at the top layer of the network. Traditional systems mostly rely on centralized cloud server storage. They easily become single-point attack targets and pose internal tampering risks. This model cleverly introduces a cloud and blockchain collaborative storage architecture. Massive business entity data is encrypted and stored in cloud servers with powerful computing capabilities. More core device identity public keys, access control policies, and signature records are distributed and anchored on the blockchain ledger. The blockchain network applies the DPoS mechanism to achieve systematic trading consistency. The decentralized and tamper-proof characteristics of the blockchain provide an additional digital trust shield for cloud data. Internal administrators of cloud servers cannot silently tamper with or delete historical data without leaving on-chain audit traces. This achieves transparency and security in data storage. The blockchain network acts in the role of lightweight information recording and cross-institutional information exchange. Note that it only verifies the IoT data transaction validity, but does not verify the signature’s validity.
- *Data Utilization*: It involves remote diagnostic analysis in hospitals and accurate automated scheduling in factories. It also involves collaborative decision-making in autonomous driving and remote control of smart homes. The core controlled verification and non-repudiation of the model demonstrate irreplaceable practical value in this application stage. Here, the receiver first verifies the validity when they receive the transmitted IoT data. Authorized institutions or systems retrieve historical data for analysis and decision-making. They must rely on blockchain records to conduct strict signature verification on the data. This ensures the utilized data sources are accurate. Real disputes may occur in certain scenarios. These include traffic accident responsibility division or medical diagnostic accident accountability. The signature mechanism makes it impossible for the data sender to deny previously issued data records or control commands. The system can accurately trace the entire process of generation, circulation, and use of every piece of data. It provides digital evidence with the highest legal validity. It effectively clarifies the responsibilities of all parties and promotes the secure operation of the entire ecosystem.

Based on the ID-US scheme, this blockchain-based model can achieve security and control. The verification requires the participation of the signer through an interactive confirmation protocol. The signer decides when, to whom, and whether verification is performed. A disavowal protocol resolves disputes when a malicious signer denies a valid signature or falsely claims one. The control over verification therefore stays with the signer. Cryptographic protocols and the blockchain jointly guarantee the binding force of this control.

### 4.2. The Proposed ID-US Scheme

This ID-US scheme integrates the identity mechanism to improve signature traceability. It mainly consists of five algorithms, Setup, KeyExt., Sign, Verify, and Confirmation/disavowal.

Setup: This ID-US is constructed by a lattice problem $ℜ-SIS_{q,n,m,\beta }^{\kappa }$ as shown in **Definition 4**, where $\beta \geq \sqrt{m}q^{n/m}$ and ${ℜ}_{q}^{n\times m}$. KGC first sets the system parameters according to the following Algorithm 1.
**Algorithm 1** Setup**Input:** ℜ−SIS, security parameter *n* **Output:** System parameter pp;
  1:Sets q=q(n)≥3 as a prime number;  2:Sets m≥3nlogq as a positive integer;  3:KGC randomly selects an approximate random distribution matrix $mpk=A\in Z_{q}^{n\times m}$;  4:Generates a basis $T\in Z_{q}^{m\times m}$ from $\Lambda^{⊥}\left(A\right)$, $\left|\right|\tilde{T}\left|\right|\leq L$;  5:Sets $H_{1},H_{2},H_{3},H_{5}:\;{\left\{0,1\right\}}^{*}\to Z_{q}^{n\times m}$ as the hash functions;  6:Sets $H_{4}:\;{\left\{0,1\right\}}^{*}\to Z_{q}^{m}$ as the other one hash function;  7:Serves the master public key mpk=A and master secret key msk=T;  8:Opens $pp=\{n,q,m,A,H_{1},H_{2},H_{3},H_{4},H_{5}\}$.

$\mathrm{KeyExt}.\;(pp,ID_{i})$: Under the system parameter pp, KGC generates the key pair (Pk,Sk) for user $ID_{i}$ according to the following Algorithm 2.
**Algorithm 2** KeyExt.**Input:** pp, and $ID_{i}$ **Output:** Keys pair (Pk,Sk)
  1:KGC calculates $a_{ID_{i}}=H_{1}\left(ID_{i}\right)\in Z_{q}^{n\times m}$;  2:Samples $s_{ID_{i}}=SampleMat\left(A,T,\sigma ,a_{ID_{i}}\right)\in Z_{q}^{m\times m}$, where $As_{ID_{i}}=a_{ID_{i}}\;modq$, $\left|\right|s_{ID_{i}}\left|\right|\leq \sigma \sqrt{m}$, and $\sigma \geq \left|\right|\tilde{T}\left|\right|w\left(\sqrt{logm}\right)$;  3:Randomly chooses $sd\in {\left\{0,1\right\}}^{*}$ as a seed;  4:Calculates $L=H_{2}\left(sd\right)\;\in Z_{q}^{n\times m}$;  5:Randomly selects $x\in D_{\sigma }^{m}$;  6:Calculates X=Lxmodq;  7:Returns $\left(Pk,Sk\right)=\{\left(a_{ID_{i}},L,X\right),\left(s_{ID_{i}},x\right)\}$ back to user $ID_{i}$.


Sign(pp,Pk,Sk,μ): Under the system parameter pp, and key pair (Pk,Sk), the user $ID_{i}$ signs message μ according to the following Algorithm 3.
**Algorithm 3** Sign**Input:** pp, (Pk,Sk), and μ **Output:** ID-US *e*
  1:Randomly chooses $y\in D_{\sigma }^{m}$;  2:Calculates $U=H_{3}\left(\mu \right)\;\in Z_{q}^{n\times m}$;  3:Calculates $e_{1}=H_{4}\left(X+Ay\;mod\;q,\;U\right)$;  4:Calculates $e_{2}=\left(y+s_{ID_{i}}e_{1}\right)\;mod\;q$;  5:Calculates $e_{3}=Ux\;mod\;q$;  6:Opens ID-US $e=(e_{1},e_{2},e_{3},\mu )$.


Verify(pp,Pk,e,μ): Given pp, *e*, and μ, the verifier can verify the validity of the ID-US *e*. Details are shown in the following Algorithm 4.
**Algorithm 4** Verify**Input:** pp, *e*, and μ **Output:** Accept or Reject
  1:Verifier first checks: if $\left|\right|e_{2}\left|\right|>T$ then  2:   Rejects  3:else Continues  4:If $\left|\right|e_{2}{\left|\right|}_{\infty }\geq q/4$ then  5:   Rejects  6:else Continues  7:If $e_{1}=H_{4}\left(X+Ae_{2}-a_{ID_{i}}e_{1}\;mod\;q,\;U\right)$  8:   Accepts


Here, $T=\eta \sqrt{m\sigma }$ is an acceptable threshold, and η is generally set within the interval [1.1,1.4].

Confirmation/Disavowal(pp,e): Given pp and *e*, the signer and verifier cooperate to confirm or deny the ID-US *e*. Details are shown in Algorithm 5.
**Algorithm 5** Confirmation/Disavowal**Input:** pp, and *e* **Output:** Confirmation or Disavowal
   1:Signer and verifier calculate *L* and *X*;   2:Chooses a permutation *r* over (1,2,…,m);   3:Chooses a vector $\pi \in D_{\sigma }^{m}$;   4:Calculates $F_{1}=H_{5}\left(r||L\pi \;mod\;2q\right)$;   5:Calculates $F_{2}=H_{5}\left(r\left(\pi \right)\right)$;   6:Calculates $F_{3}=H_{5}\left(r\left(\pi +x\right)\right)$;   7:Calculates $F_{4}=H_{5}\left(r||U\pi \;mod\;2q\right)$;   8:Sets the commitments $\left(F_{1},F_{2},F_{3},F_{4}\right)$;   9:Verifier sends a challenge f∈{1,2,3} to signer; 10:Signer answers (r,π) for f=1 challenge; 11:Answers (r,π+x) for f=2 challenge; 12:Answers (r(π),r(x)) for f=3 challenge; 13:Verifier checks the validity of $F_{1}$, $F_{2}$, and $F_{4}$ for f=1 challenge; 14:Checks the validity of $F_{1}$, $F_{2}$, and $F_{4}$ for f=2 challenge; 15:If $F_{4}=H_{5}(r||U\left(\pi +x\right)-e_{3})$, outputs confirmation; 16:else $F_{4}\neq H_{5}(r||U\left(\pi +x\right)-e_{3})$, outputs disavowal; 17:Checks the validity of $F_{2}$, $F_{3}$, and $\left||r\left(e\right)|\right|\leq \beta \sqrt{m}$ for f=3 challenge; 18:Outputs “Confirmation” when verifications are all successful; 19:Otherwise, outputs “Disavowal”.


## 5. Security Analysis

This section first analyzes the correctness of the ID-US scheme, and then proves its soundness and unforgeability.

### 5.1. Correctness

When $\left|\right|e_{2}\left|\right|\leq T$ and $\left|\right|e_{2}{\left|\right|}_{\infty }<q/4$, the ID-US $e=(e_{1},e_{2},e_{3},\mu )$ can pass the verification as shown in Algorithm 4. Then, it has Equation (4).

(4) $$H_{4}\left(X+Ae_{2}-a_{ID_{i}}e_{1}\;mod\;q,\;U\right)=H_{4}\left(X+Ay\;mod\;q,U\right)$$

The former Equation (4) holds when $X+Ae_{2}-a_{ID_{i}}e_{1}=X+Ay\;mod\;q$ holds, and the details are shown in Equation (5).

(5) $$\begin{array}{cc} X+Ae_{2}-a_{ID_{i}}e_{1} & =X+A\left(y+s_{ID_{i}}e_{1}\right)-a_{ID_{i}}e_{1}\\ \; & =X+Ay+As_{ID_{i}}e_{1}-a_{ID_{i}}e_{1}\\ \; & =X+Ay+a_{ID_{i}}e_{1}-a_{ID_{i}}e_{1}\\ \; & =X+Ay\;mod\;q \end{array}$$

### 5.2. Soundness

**Theorem** **1.** ***Soundness*** *. For a valid or invalid (π,e), it always outputs Prob(Disavowal)=1 or Prob(Confirmation)=1, respectively.*

**Proof.**  Here, the valid and invalid (π,e) are analyzed separately.

(1) *Invalid case*: The equation $e_{3}\neq Ux^{T}\;mod\;q$ holds for an invalid pair (π,e). Then, the signer responds according to the following principle.

- If f=1, responds with $\psi_{1}$ and $\theta_{1}$;
- If f=2, responds with $\psi_{2}$ and $\theta_{2}$;
- If f=3, responds with $\theta_{3}$ and $\theta_{3}$.

Then, the verifier has:

(6) $$\left\{\begin{array}{c} F_{1}=H_{5}(\psi_{1}||L\theta_{1}^{T}\;mod\;q)=H_{5}(\psi_{2}\left|\right|\left(L\theta_{2}^{T}-X\right)\;mod\;q)\\ F_{2}=H_{5}\left(\psi_{1}\left(\theta_{1}\right)\right)=H_{5}\left(\theta_{4}\right)\\ F_{3}=H_{5}\left(\psi_{s}\left(\theta_{2}\right)\right)=H_{5}\left(\theta_{3}+\theta_{4}\right)\\ F_{4}=H_{5}(\psi_{1}||U\theta_{1}^{T}\;mod\;q)=H_{5}(\psi_{2}\left|\right|\left(U\theta_{2}^{T}-e_{3}\right)\;mod\;q) \end{array}\right.$$

Unfortunately, $H_{5}$ has a collision, or the results in Equation (7) hold by ${\left||\theta |\right|}_{3}\leq \beta \sqrt{m}$.

(7) $$\left\{\begin{array}{c} \psi_{1}=\psi_{2}\\ L\theta_{1}^{T}\;mod\;q=\left(L\theta_{2}^{T}-X\right)\;mod\;q\\ \phi_{1}\left(\theta_{1}\right)=\theta_{4}\\ \phi_{2}\left(\theta_{2}\right)=\theta_{3}+\theta_{4}\\ U\theta_{1}^{T}\;mod\;q=\left(U\theta_{2}^{T}-e_{3}\right)\;mod\;q \end{array}\right.$$

Next, it has $\theta_{2}-\theta_{1}=\psi_{1}^{-1}\left(\theta_{3}\right)$ from the second and third equations in Equation (9). It also has $X=L\psi_{1}^{-1}{\left(\theta_{3}\right)}^{T}$. So, $x=\psi_{1}^{-1}\left(\theta_{3}\right)$ holds. Then, it has $e_{3}=U\psi_{1}^{-1}{\left(\theta_{3}\right)}^{T}\;mod\;q=Ux^{T}\;mod\;q$. However, $e_{3}\neq Ux^{T}\;mod\;q$ holds for an invalid (π,e). This leads to a contradiction, and it shows that the signer cannot correctly answer the former three challenges at the same time. Assuming that the success probability of the signer correctly answering each challenge f=1 (or f=2, f=3) is $\frac{1}{2}$, the success probability of answering three challenges simultaneously is $\frac{1}{2}*\frac{1}{2}*\frac{1}{2}=\frac{1}{8}$. Therefore, $Prob\left(Disavowal\right)=1-{\left(\frac{1}{8}\right)}^{N}$ will be close to 1 with increasing N(N≥2), where *N* is the response times.

(2) *Valid case*: The equation $e_{3}=Ux^{T}\;mod\;q$ holds for a valid pair (π,e). As in the former analysis in the *Invalid case*, the success probability of answering three challenges simultaneously is $\frac{1}{8}$. Therefore, $Prob\left(Confirmation\right)=1-{\left(1-1/8\right)}^{N}$ will be close to 1 with increasing *N*, where *N* is the response times. □

### 5.3. Unforgeability

**Theorem** **2.** ***Unforgeability*** *: Under the lattice assumption, this ID-US cannot be forged by an adversary with a chosen-message attack in the ROM.*

**Proof.**  In the ROM, one challenger *C* and one adversary *E* execute a query-response game. *E* performs the query, but it does not query on the target message $\mu^{*}$. *C* responds to *E*’s queries. When *E* obtains enough information, it attempts to forge an ID-US. Next, *C* attempts to solve the $Z-SIS_{q,n,m,\beta }^{\kappa }$ instance $A^{\prime }v=0\;mod\;q$. Details are as follows.

- *Initialize*: *C* initiates and opens the system parameters pp.
- *$H_{1}$ query*: For $H_{1}$, *E* performs the query on a random user $ID_{i}$. *C* sets a list $List_{H_{1}}$ to store the query results. When it obtains the query, it first checks $List_{H_{1}}$ and sends $\left(a_{ID_{i}},ID_{i}\right)$ back to *E* if the query is queried. If it does not exist, it calculates $a_{ID_{i}}=H_{1}\left(ID_{i}\right)$, sends the result $\left(a_{ID_{i}},ID_{i}\right)$ to *E*, and records it in $List_{H_{1}}$.
- *$H_{2}$ query*: For $H_{2}$, *E* performs the query on a random seed sd. *C* sets a list $List_{H_{2}}$ to store the query results. When it obtains the query, it first checks $List_{H_{2}}$ and sends (sd,L) back to *E* if the query is queried. If it does not exist, it calculates $L=H_{2}\left(sd\right)$, sends the result (sd,L) to *E*, and records it in $List_{H_{2}}$.
- *$H_{3}$ query*: For $H_{3}$, *E* performs the query on a non-target message μ. *C* sets a list $List_{H_{3}}$ to store the query results. When it obtains the query, it first checks $List_{H_{3}}$ and sends (μ,U) back to *E* if the query is queried. If it does not exist, it calculates $U=H_{3}\left(\mu \right)$, sends the result (μ,U) to *E*, and records it in $List_{H_{3}}$.
- *$H_{4}$ query*: For $H_{4}$, *E* performs the query on a non-target message μ. *C* sets a list $List_{H_{4}}$ to store the query results. When he obtains the query, he first checks $List_{H_{4}}$ and sends $\left(e_{1},U\right)$ back to *E* if the query is queried. If it does not exist, he performs the $H_{2}$ query to obtain *U* firstly, calculates $e_{1}=H_{4}\left(X+Ay\;mod\;q,U\right)$, sends the result $\left(e_{1},U\right)$ to *E*, and records it in $List_{H_{4}}$.
- *Secret key query*: For secret key, *E* performs the query on a non-target user $ID_{i}^{\prime }$. *C* sets a list $List_{SK}$ to store the query results. When it obtains the query, it first checks $List_{SK}$ and sends $\left(s_{ID_{i}^{\prime }},a_{ID_{i}^{\prime }}\right)$ back to *E* if the query is queried. If it does not exist, it performs the $H_{1}$ query to obtain $a_{ID_{i}^{\prime }}$ firstly, calculates $s_{ID_{i}^{\prime }}=SampleMat\left(A,T,\sigma ,a_{ID_{i}^{\prime }}\right)$, sends the result $\left(s_{ID_{i}^{\prime }},a_{ID_{i}^{\prime }}\right)$ to *E*, and records it in $List_{SK}$.
- *Signature query*: For signature, *E* performs the query on a non-target message μ. *C* sets a list $List_{S}$ to store the query results. When it obtains the query, it first checks $List_{S}$ and sends (e,μ) back to *E* if the query has been queried. If it does not exist, it performs the $H_{3}$ query to obtain *U* firstly, calculates $e_{1}=H_{4}\left(X+Ay\;mod\;q,\;U\right)$, $e_{2}=\left(y+s_{ID_{i}^{\prime }}e_{1}\right)\;mod\;q$, and $e_{3}=Ux\;mod\;q$. Then, it sends the result $\left(e=\left(e_{1},e_{2},e_{3}\right),\mu \right)$ to *E*, and records it in $List_{S}$.
- *Forge*: Under the former assumption and queried information, *E* can forge a valid ID-US $\left(e^{\prime }=\left(e_{1}^{\prime },e_{2}^{\prime },e_{3}^{\prime }\right),\mu^{\prime }\right)$ for the target message $\mu^{\prime }$, and return it to *C*.
- *Challenge*: *C* generates a signature $\left(e^{\prime \prime }=\left(e_{1}^{\prime \prime },e_{2}^{\prime \prime },e_{3}^{\prime \prime }\right),\mu^{\prime }\right)$ for the same message $\mu^{\prime }$. Next, it utilizes these two valid signatures $e^{\prime }$ and $e^{\prime \prime }$ to solve the $Z-SIS_{q,n,m,\beta }^{\kappa }$ instance. Firstly, it computes $U^{\prime }=H_{3}\left(\mu^{\prime }\right)$, and obtains the following results, as shown in Equation (8).
  (8) $$\left\{\begin{array}{c} e_{1}^{\prime }=H_{4}\left(X+Ay^{\prime }\;mod\;q,\;U^{\prime }\right)\\ e_{1}^{\prime \prime }=H_{4}\left(X+Ay^{\prime \prime }\;mod\;q,\;U^{\prime }\right) \end{array}\right.$$
- *Analyze*: Here, it has $Ay^{\prime }=A\left(e_{2}^{\prime }-s_{ID_{i}}^{\prime }e_{1}^{\prime }\right)=Ae_{2}^{\prime }-a_{ID_{i}}^{\prime }e_{1}^{\prime }$ as $e_{2}^{\prime }=\left(y^{\prime }+s_{ID_{i}}^{\prime }e_{1}^{\prime }\right)\;mod\;q$ holds. Therefore, it has the following results in Equation (9).
  (9) $$\left\{\begin{array}{c} e_{1}^{\prime }=H_{4}\left(X+Ay^{\prime }\;mod\;q,\;U^{\prime }\right)=H_{4}\left(X+Ae_{2}^{\prime }-a_{ID_{i}}^{\prime }e_{1}^{\prime }\;mod\;q,\;U^{\prime }\right)\\ e_{1}^{\prime \prime }=H_{4}\left(X+Ay^{\prime \prime }\;mod\;q,\;U^{\prime }\right)=H_{4}\left(X+Ae_{2}^{\prime \prime }-a_{ID_{i}}^{\prime }e_{1}^{\prime \prime }\;mod\;q,\;U^{\prime }\right) \end{array}\right.$$
  Next, it also has Equation (10).
  (10) $$\left\{\begin{array}{c} X+Ay^{\prime }=X+Ae_{2}^{\prime }-a_{ID_{i}}^{\prime }e_{1}^{\prime }\;mod\;q\\ X+Ay^{\prime \prime }=X+Ae_{2}^{\prime \prime }-a_{ID_{i}}^{\prime }e_{1}^{\prime \prime }\;mod\;q \end{array}\right.$$
  Then, it also has Equation (11).
  (11) $$\left\{\begin{array}{c} A\left(e_{2}^{\prime }-y^{\prime }\right)=a_{ID_{i}}e_{1}^{\prime }\;mod\;q\\ A\left(e_{2}^{\prime \prime }-y^{\prime \prime }\right)=a_{ID_{i}}e_{1}^{\prime \prime }\;mod\;q \end{array}\right.$$
  Here, from Equation (11), it has $A\left(e_{2}^{\prime }-e_{2}^{\prime \prime }-y^{\prime }+y^{\prime \prime }\right)=a_{ID_{i}}\left(e_{1}^{\prime }-e_{1}^{\prime \prime }\right)\;mod\;q$. Because $e_{1}^{\prime }\neq e_{1}^{\prime \prime }$, $a_{ID_{i}}\in Z_{q}^{n\times m}$, and $e_{1}^{\prime },e_{q}^{\prime \prime }\in Z_{q}^{m}$, it has $A(e_{2}^{\prime }-e_{2}^{\prime \prime }-y^{\prime }+y^{\prime \prime })=0\;mod\;q$. So $v=e_{2}^{\prime }-e_{2}^{\prime \prime }-y^{\prime }+y^{\prime \prime }$ is a solution of the $Z-SIS_{q,n,m,\beta }^{\kappa }$ instance Av=0modq. However, it cannot solve the SIS problem with the current most advanced computing conditions, so this solution is not credible. It also indicates that the assumption of *E* is not correct, and *E* cannot forge a valid ID-US. Meanwhile, the probability that *E* can forge a valid ID-US is $\frac{1}{q_{H_{1}}+q_{H_{2}}+q_{H_{3}}+5/4\left(q_{H_{4}}\right)+q_{SK}+5/4\left(q_{S}\right)}$. Here, $q_{H_{1}}$, $q_{H_{2}}$, $q_{H_{3}}$, $q_{H_{3}}$, $q_{SK}$, and $q_{S}$ are the query times as *E* can perform enough queries when it obtains enough information. The factor 5/4 means that we assume the existence of a 1/4 probability of performing $H_{2}$ and $H_{3}$ queries in $H_{4}$ and Signaturequery, respectively. However, with the query times increasing, *E*’s success probability will become negligible. Therefore, this ID-US scheme can capture the security property of unforgeability.

□

### 5.4. Other Security Properties

Some other security properties achieved by the proposed ID-US scheme are analyzed in this subsection. These security properties are obtained by function design and do not require a formal security proof with ROM. Following are the detailed analyses.

- *Non-repudiation*: There are two settings that can guarantee this property of non-repudiation. If the signature has passed verification, the signer cannot deny it, as the confirmation/disavowal mechanism can prevent this repudiation. Meanwhile, the signer’s identity is inserted into the signature, and the signer cannot deny a valid signature with his own identity.
- *Post-quantum security*: This ID-US is constructed with the lattice assumption, which can provide anti-quantum-attack security for IoT data sharing across different IoT devices and networks.
- *Controlled verification*: The proposed ID-US scheme can guarantee that a valid signature cannot be denied by the signer. The confirmation/disavowal mechanism utilizes a challenge form to achieve non-repudiation. Only after passing the challenges can the signature be accepted by the verifier. This controlled verification guarantees the security of user privacy and IoT data.
- *Data anonymity*: The controlled verification model can also be established with blockchain technology. The real data is stored in the native server, and the lightweight messages, such as storage address, operation records, and transaction information, are recorded in the blockchain ledger. Only after passing the signature verification can the receiver obtain the real data from the storage address. This form saves online ledger space but also guarantees data anonymity.

In a word, the results of correctness analysis, security proof, and other related security analysis show that this ID-US scheme is correct and secure for data sharing in IoT networks.

## 6. Performance Evaluations

### 6.1. Scyther Test

**Figure 2:**
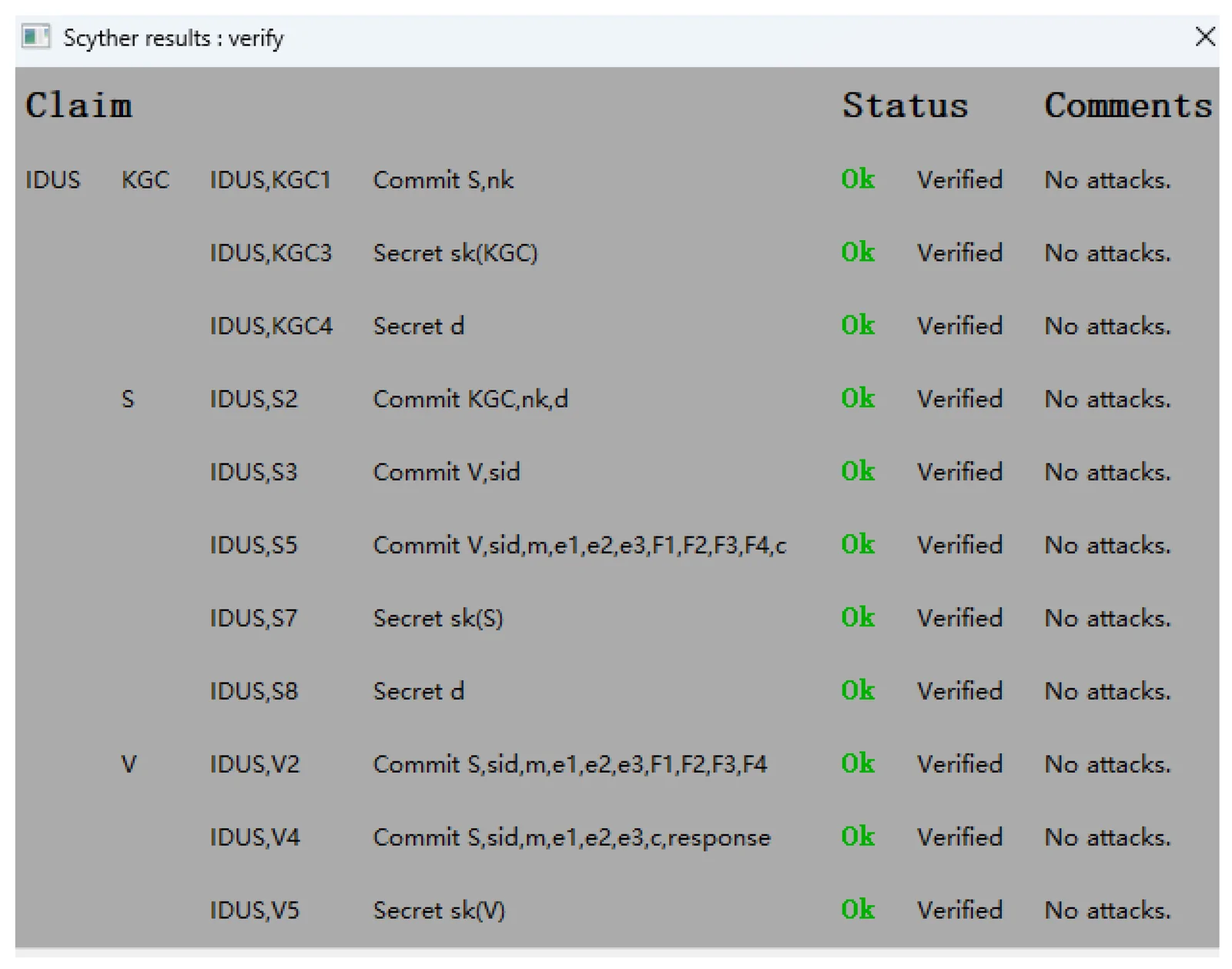
Scyther test results.

The Scyther verification results (as shown in Figure 2) indicate that this ID-US scheme achieves complete security under all analyzed protocol roles. Specifically, the KGC, the Signer (S), and the Verifier (V) all report “No attacks,” with every security claim successfully verified. The following paragraphs present a detailed analysis of these results.

- The KGC’s behavior is verified, which asserts the secrecy of the commitment, the KGC’s master secret key sk, and the secret seed sd. All three claims are marked as “Ok Verified,” confirming that the KGC protocol role maintains the confidentiality of all sensitive parameters throughout execution. This indicates that the master key and the randomly sampled secret seed sd are not leaked to the adversary through any observable communication channel, even under the assumption of a Dolev–Yao attacker with full control over the network.
- The signer’s behavior is verified. The commitments are all verified as secure, confirming that the signer faithfully follows the protocol and that no sensitive intermediate values are disclosed in the commitment phase. Furthermore, the claims also confirm the secrecy of the signer’s private key sk and the secret value sd. The successful verification of these claims demonstrates that the signing process preserves the privacy of the signer’s long-term private key and all ephemeral random values, which is essential for ensuring unforgeability and key-isolating security in lattice-based identity-based signatures.
- The verifier’s behavior is verified. The commitments are verified as secure, indicating that the verifier correctly preserves the integrity of the protocol transcripts. Moreover, the secrecy of the verifier’s private key sk is also confirmed. This demonstrates that the ID-US scheme maintains the confidentiality of the verifier’s credentials, which is a critical requirement for achieving the deniable/undeniable property of the scheme—namely, that the verifier cannot fabricate valid transcripts to later falsely attribute signatures to the signer.

In total, these test results show that this ID-US has no attack weak points from the perspectives of KGC, signer, and verifier, so it is correct and secure.

### 6.2. Efficiency Analysis

This section first analyzes the efficiency of the proposed ID-US with similar schemes of Li and He et al. 2024 [7], Li and Shen et al. 2023 [8], Chen et al. 2026 [9], Dey et al. 2024 [36], Almende et al. 2025 [37] published in recent years. Then, the performance of the proposed model and ID-US scheme is evaluated.

#### 6.2.1. Efficiency Comparison

The comparisons of key sizes for Pk, Sk, and Signature are presented in Table 1. This table applies the same comparison format and unifies the system parameters. The items of this ID-US scheme have certain advantages, as the Pk is smaller than that in schemes [7,8,9], the Sk is smaller than those in schemes [7,36], and the Signature is slightly smaller than that in schemes [8,9]. The smaller key size can save storage space and improve the processing speed for data sharing among different IoT devices. More importantly, this ID-US scheme has an identity mechanism that can provide a traceable mechanism for transaction disputes.

Meanwhile, the time cost comparisons for KeyGen., Sign, Verify, and Confirm./Disav. are presented in Table 2. Here, the same comparison form is also applied. $T_{MMV}$ is the multiplication of a matrix and a vector, $T_{MMM}$ is the multiplication of two matrices, $T_{AVV}$ is the addition of two vectors, $T_{AMM}$ is the addition of two matrices, $T_{H}$ is the hash algorithm (SHA 256), $T_{Trap}$ is the trapdoor, and $T_{Samp}$ is the SamplePre. These items influence the execution time of the ID-US scheme. Less execution time will improve the transaction processing time. The items of this ID-US scheme have certain advantages.

#### 6.2.2. Performance Evaluation

To clearly show the differences between this ID-US and similar schemes, the performance is evaluated on a Windows 11 desktop with an Intel Core i7-9700 CPU at 3.2 GHz and 16 GB RAM.

**Figure 3:**
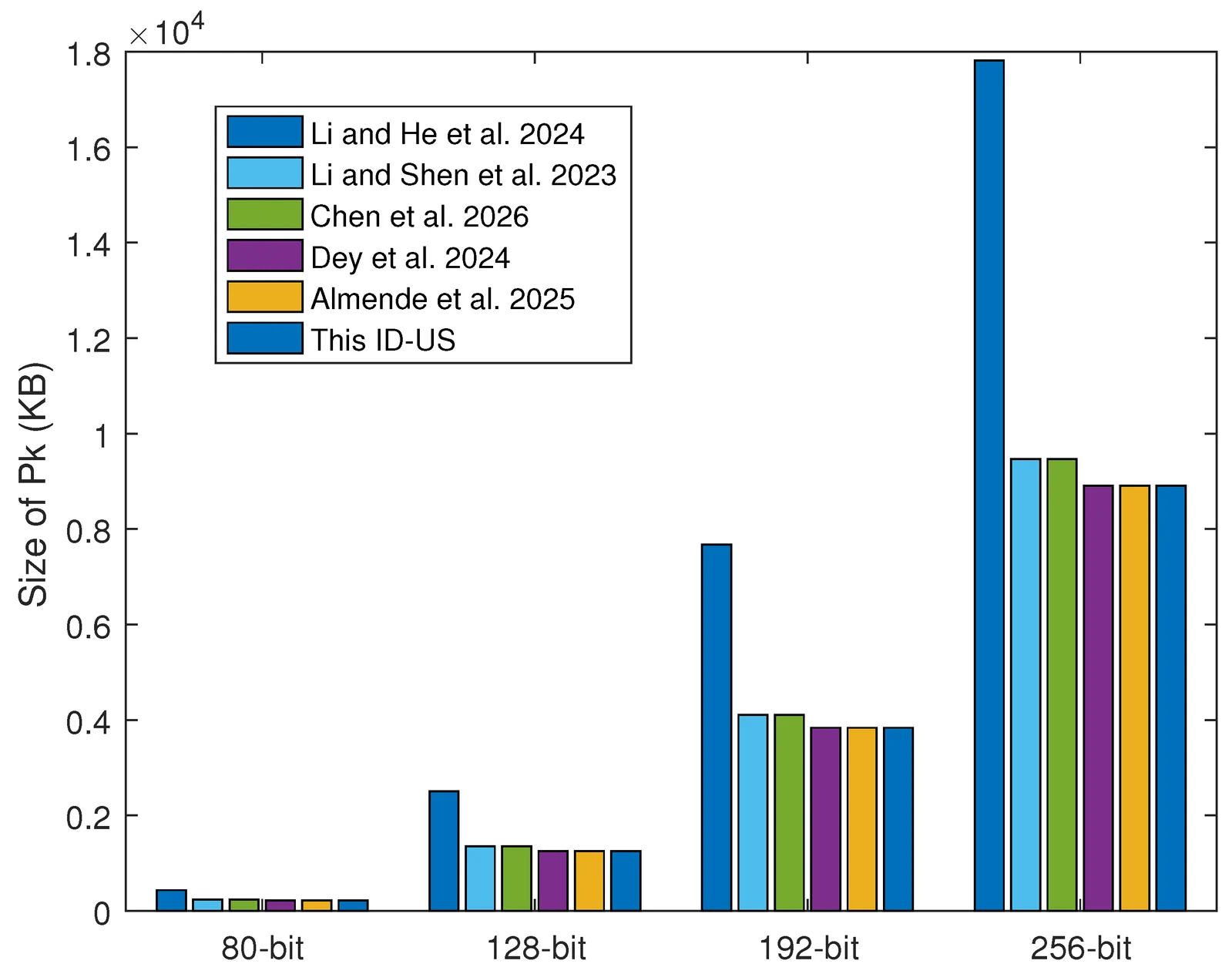
Pk size comparison [7,8,9,36,37].

**Figure 4:**
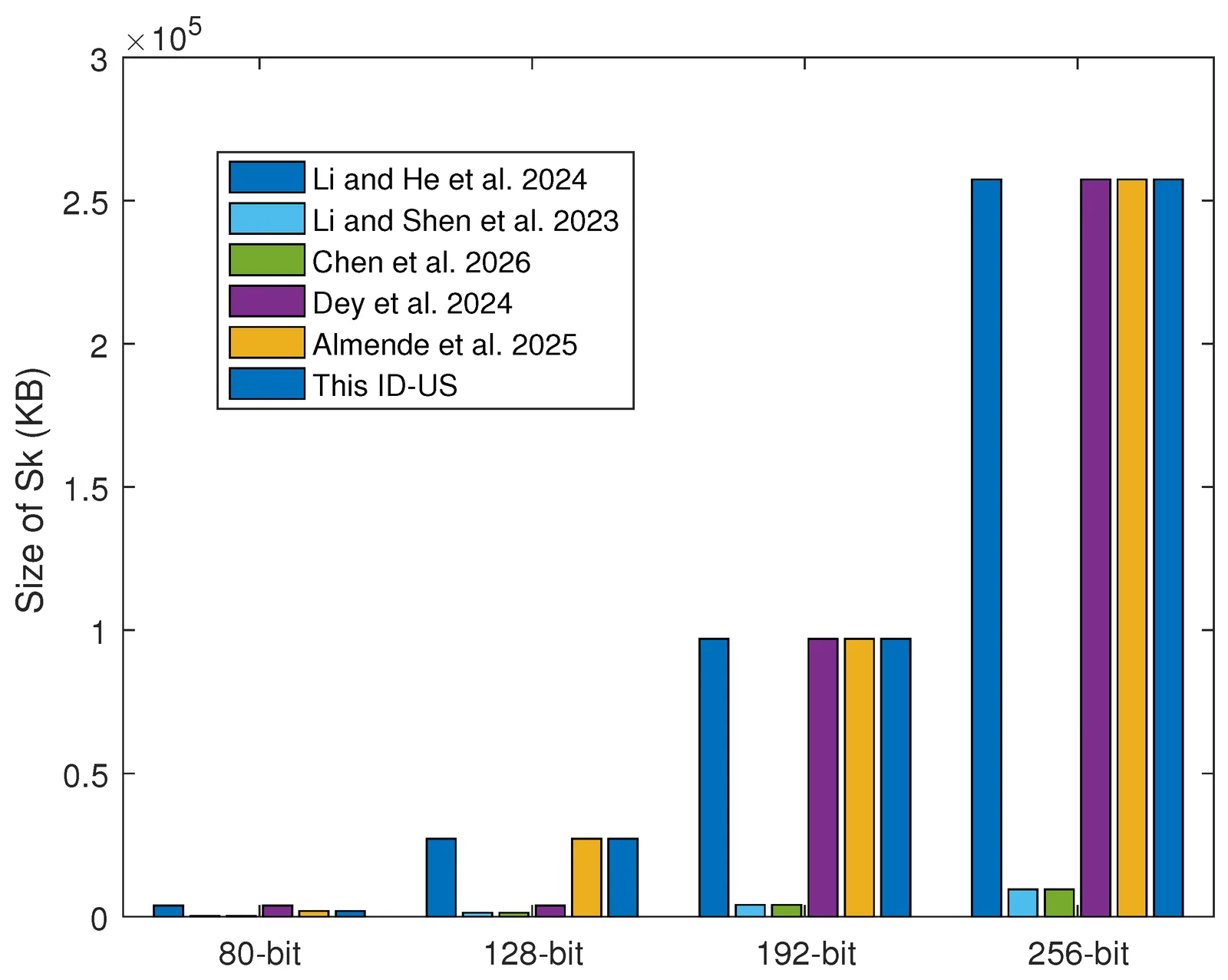
Sk size comparison [7,8,9,36,37].

**Figure 5:**
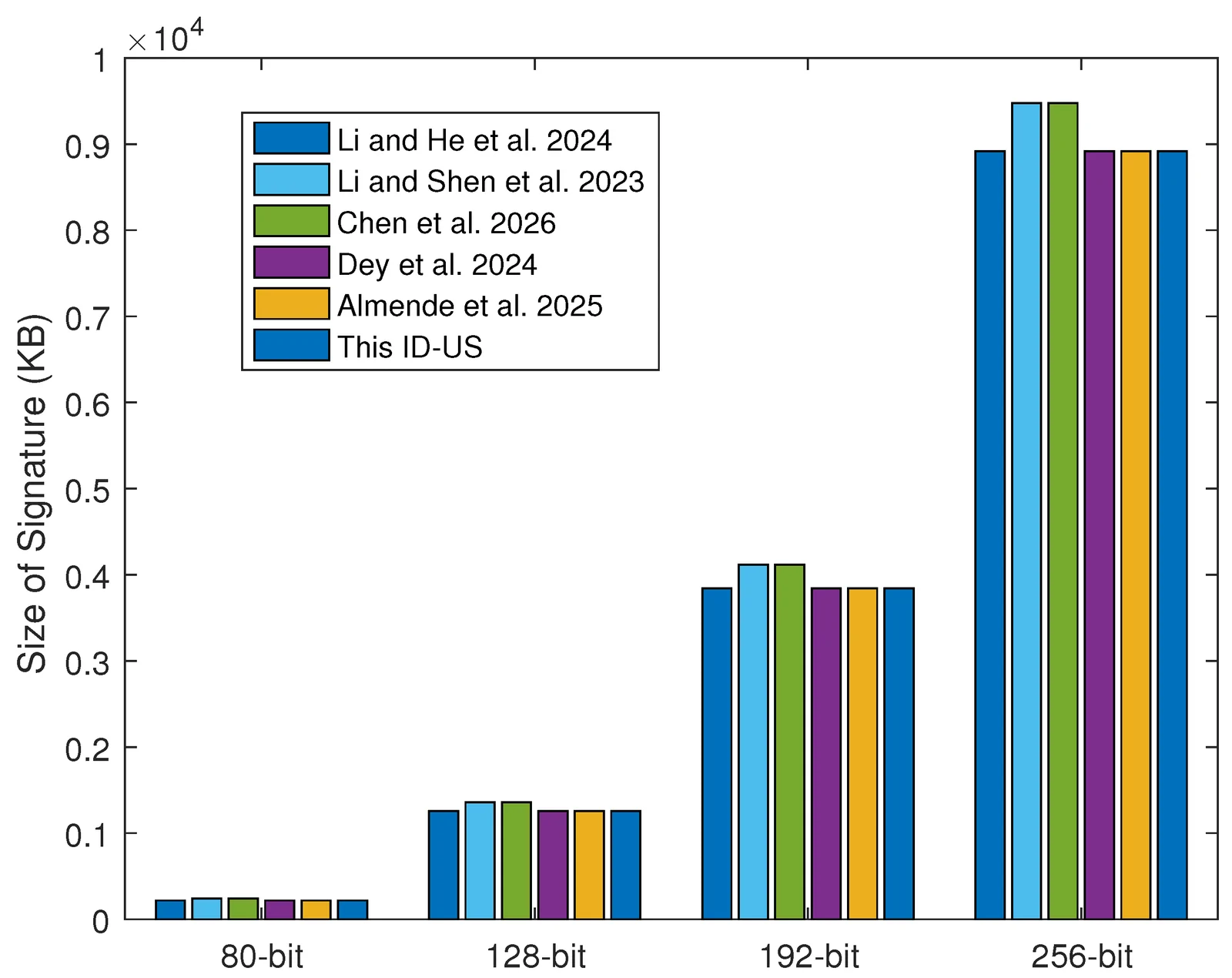
Signature size comparison [7,8,9,36,37].

As shown in Figure 3, Figure 4 and Figure 5, the key size performances are presented. Here, four security levels are selected, which use the same settings as in [9]. The 80-bit level is with the system parameters n=256, m=2312, and $q=2^{10}$, 128-bit is with the system parameters n=512, m=5549, and $q=2^{12}$, 192-bit is with the system parameters n=768, m=9710, and $q=2^{14}$, and 256-bit is with the system parameters n=1024, m=14796, and $q=2^{16}$. Here, the 80/128/192-bit security corresponds to the security level of classical encryption. The results show that this ID-US scheme has certain advantages over similar schemes. These performance results are consistent with the theoretical analysis as shown in Table 1.

**Figure 6:**
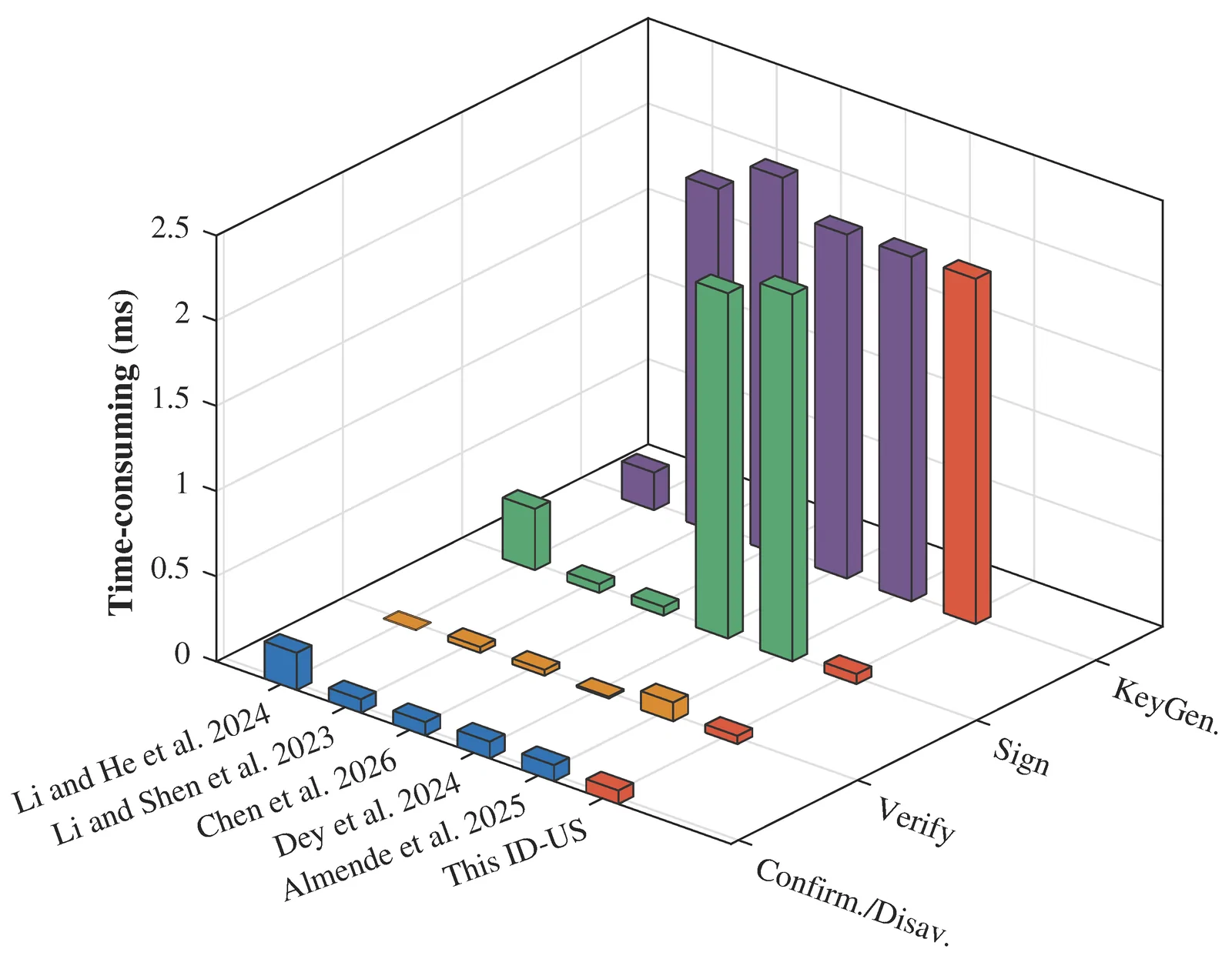
Time consumption comparison [7,8,9,36,37].

In Figure 6, the performance of time costs is presented. Here, the time consumption of every operation item is executed in the aforementioned environment. $T_{MMV}=0.01$ ms, $T_{MMM}=0.1$ ms, $T_{AVV}=0.003$ ms, $T_{AMM}=0.01$ ms, $T_{H}=0.008$ ms, $T_{Trap}=2$ ms, and $T_{Samp}=2$ ms, which are set by performing 100 calculations in the aforementioned environment and averaging the results. The execution results are shown in Table 3. For KeyGen., the time costs remain at the same level, although it uses the Sample algorithm to generate the private key. For Sign, the time costs have a big advantage over those in schemes [7,36,37] as there is no need for $T_{Trap}$ and $T_{Samp}$ algorithms with high time consumption. For verification, the time costs have a big advantage over those in scheme [37]. For the Confirm./Disav., the time costs have a big advantage over those in schemes [7,36,37]. These performance results are consistent with the theoretical analysis as shown in Table 2. Therefore, the proposed ID-US can support more efficient transaction verification and IoT data sharing.

### 6.3. Energy Consumption and Transaction Latency

**Figure 7:**
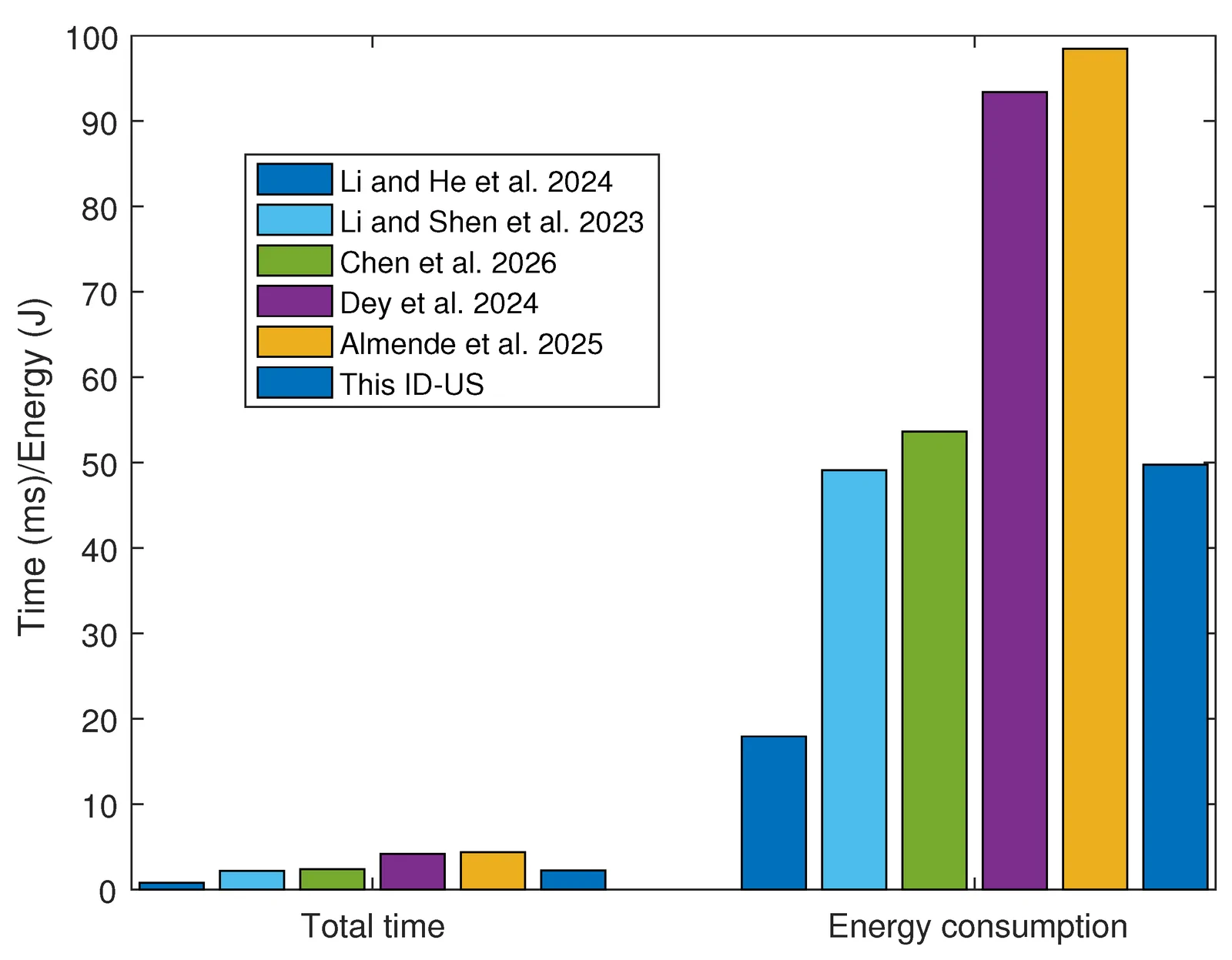
Energy comparison [7,8,9,36,37].

To prove the application ability of the proposed ID-US scheme, an IoT device with the configuration of 3.0 V and 7.5 mA is selected. The total time consumption is composed of four algorithms: KeyGen., Sign, Verify, and Confirm./Disav. For example, the total time consumption of this ID-US scheme is 2.212 ms, so the energy consumption is 2.212∗3.0∗7.5=49.77 J. With the same IoT device, the other five related schemes are also computed, and the comparison results are shown in Figure 7.

**Figure 8:**
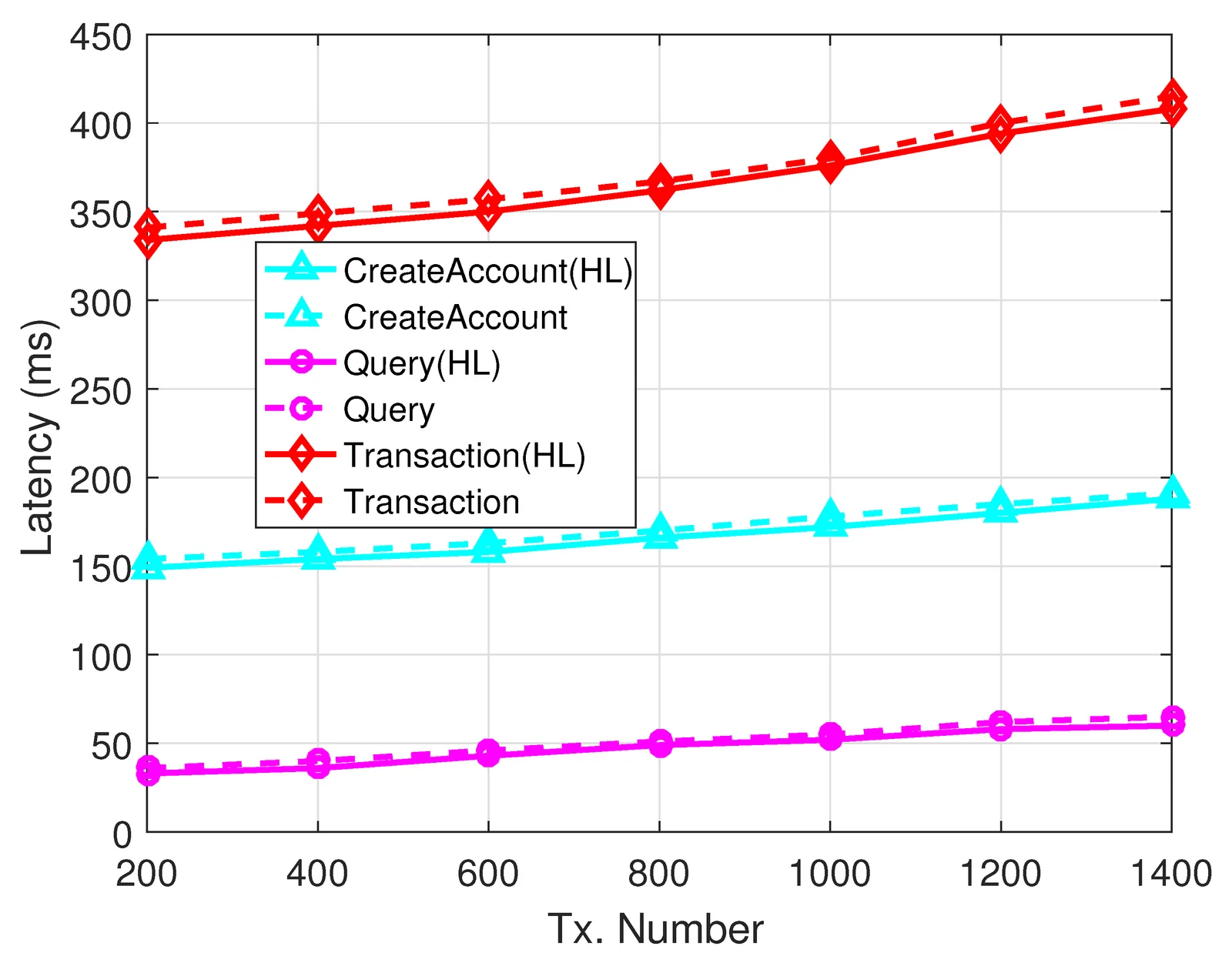
Latency comparison.

To verify the effectiveness of the proposed controlled verification model, the transaction latency has been measured and compared with Hyperledger (HL). It simulates five IoT nodes, and the transaction (Tx.) number increases from 200 to 1400. For the transaction throughput, all the test transactions are correctly verified, packaged, and recorded. The main difference is the transaction latency, which reflects the efficiency of IoT data transaction execution. Three items of “CreateAccount”, “Query”, and “Transaction” are selected. Then, the simulation results are shown in Figure 8. Here, the dotted and solid lines represent the proposed controlled verification model and HL, respectively. Although the controlled verification model is equipped with the post-quantum lattice-based signature scheme, it does not influence the transaction latency compared with HL. Therefore, the proposed controlled verification model can maintain low transaction latency while supporting secure transactions.

## 7. Conclusions

This work utilizes the blockchain and ID-US scheme to establish a controlled verification model for IoT networks. The blockchain technology helps to establish a secure IoT data-sharing channel, and the distributed ledger also guarantees IoT data immutability and message non-loss. Meanwhile, this ID-US scheme applies an undeniable mechanism to achieve message undeniability, and the identity mechanism to guarantee message traceability. It can also provide anti-quantum capability for IoT networks in the near-future quantum computer age. Moreover, the security proof in ROM shows that this ID-US can obtain the security properties of soundness and unforgeability. In addition, this ID-US scheme has no attack weak points in the Scyther test, and the efficiency comparison results show that it has certain advantages over similar schemes in key size, time consumption, and transaction latency. Therefore, the proposed controlled verification model and ID-US scheme are suitable for protecting IoT data sharing through different IoT devices and networks.

In the future, effort will focus on the practical application of this ID-US. Meanwhile, solution improvement and performance optimization with resource-constrained IoT devices with related post-quantum signature algorithms will also be carried out.

## Figures and Tables

**Table 1:** Key size comparison.

Items	Pk	Sk	Signature
Li and He et al. 2024 [7]	2mnlogq	$2m^{2}\log q$	(m+n+mn)logq
Li and Shen et al. 2023 [8]	mnlog2q	mnlog2q	(m+n+mn)log2q
Chen et al. 2026 [9]	mnlog2q	mnlog2q	(m+n+mn)log2q
Dey et al. 2024 [36]	mnlogq	$(2m^{2}+1)\log q$	(m+n+mn)logq
Almende et al. 2025 [37]	mnlogq	$m^{2}\log q$	(m+n+mn)logq
This ID-US	mnlogq	$m^{2}\log q$	(m+n+mn)logq

**Table 2:** Time costs comparison.

Items	KeyGen.	Sign	Verify	Confirm./Disav.
Li and He et al. 2024 [7]	$2T_{MMM}+2T_{AMM}$	$2T_{H}+3T_{MMM}+2T_{MMV}+2T_{AMM}+T_{AVV}$	–	$T_{H}+2T_{MMM}+T_{AMM}$
Li and Shen et al. 2023 [8]	$T_{Trap}+T_{H}+T_{MMV}$	$2T_{H}+3T_{MMV}+2T_{AVV}$	$T_{H}+2T_{MMV}+2T_{AVV}$	$5T_{H}+3T_{MMV}+3T_{AVV}$
Chen et al. 2026 [9]	$T_{Trap}+T_{H}+T_{MMV}+2T_{MMM}$	$2T_{H}+3T_{MMV}+2T_{AVV}$	$T_{H}+2T_{MMV}+2T_{AVV}$	$5T_{H}+3T_{MMV}+3T_{AVV}$
Dey et al. 2024 [36]	$T_{Trap}+T_{H}+T_{MMV}$	$2T_{H}+T_{Samp}+T_{MMV}$	$T_{MMV}$	$4T_{H}+4T_{MMV}+2T_{AMM}+3T_{AVV}$
Almende et al. 2025 [37]	$T_{Trap}+T_{H}+T_{MMV}+T_{AVV}$	$2T_{H}+T_{Samp}+2T_{MMV}+T_{MMM}+T_{AMM}+2T_{AVV}$	$T_{MMM}+T_{AMM}$	$5T_{H}+3T_{MMV}+8T_{AVV}$
This ID-US	$T_{Samp}+2T_{H}+T_{MMV}$	$2T_{H}+3T_{MMV}+T_{AVV}+T_{AMM}$	$T_{H}+2T_{MMV}+2T_{AMM}$	$5T_{H}+3T_{MMV}+3T_{AVV}$

**Table 3:** Time costs execution comparison.

Items	KeyGen.	Sign	Verify	Confirm./Disav.
Li and He et al. 2024 [7]	0.22	0.359	–	0.218
Li and Shen et al. 2023 [8]	2.018	0.052	0.034	0.079
Chen et al. 2026 [9]	2.218	0.052	0.034	0.079
Dey et al. 2024 [36]	2.018	2.026	0.01	0.098
Almende et al. 2025 [37]	2.021	2.152	0.11	0.094
This ID-US	2.026	0.059	0.048	0.079

## Data Availability

The original contributions presented in this study are included in this article. Further inquiries can be directed to the corresponding author.
